# A randomized, phase Ib trial of recombinant human serum albumin in cirrhotic patients with ascites

**DOI:** 10.1007/s12072-025-10871-x

**Published:** 2025-07-23

**Authors:** Xinrui Wang, Wanyu Li, Fei Kong, Xiaolin Guo, Qinglong Jin, Runping Gao, Yulin Hu, Yanjun Cai, Guijie Xin, Huifan Ji, Hongxin Piao, Zhaoxu Fu, Yifei Wang, Zhiyong Piao, Siqi Wang, Rui Hua, Xiaoyu Wen, Yue Qi, Jinglan Jin, Chong Wang, Zhongfeng Wang, Fang Xu, Qiang Zhou, Xu Li, Ge Yu, Yang Wang, Tao Yang, Wei Xiang, Yu Pan, Junqi Niu, Yanhang Gao

**Affiliations:** 1 Department of Hepatology, Center of Infectious Diseases and Pathogen Biology, The First Hospital of Jilin University, Changchun, 130021 China; 2 Jilin Provincial Key Laboratory of Metabolic Liver Diseases, Jilin University, Changchun, 130021 China; 3 China-Singapore Belt and Road Joint Laboratory On Liver Disease Research, Changchun, 130021 China; 4 Yanbian Hospital of Yanbian University, Yanbian, 133000 Jilin China; 5 Department of Gastroenterology, Tonghua Central Hospital, Tonghua, 134000 Jilin China; 6 Tonghua Anrate Biopharmaceutical, Tonghua, 134000 Jilin China

**Keywords:** Recombinant human serum albumin, Safety, Efficacy, Liver cirrhosis, Phase I clinical trial

## Abstract

**Background:**

Recombinant human serum albumin (rHA) is a promising alternative to human serum albumin (HSA) for managing ascites in cirrhotic patients. This phase Ib study aims to assess the safety, tolerability, and pharmacokinetics/pharmacodynamics (PK/PD) profiles of rHA in this population.

**Methods:**

This randomized, open-label, phase Ib trial was conducted between December 2019 and September 2020 at 3 medical centers in China. Patients with cirrhotic ascites were randomly assigned to receive rHA or HSA at 10 g/day, 20 g/day, or 30 g/day. Each group had 12 participants (nine receiving rHA and three receiving HSA as positive control). Treatment lasted up to 14 days or until serum albumin levels reached 35 g/L, followed by a 28-day follow-up. Adverse events monitored assessed safety and tolerability, while PK/PD was evaluated by tracking serum albumin levels and plasma colloid osmotic pressure (PCOP) before and after each dose (ClinicalTrials.gov No. NCT04701697).

**Results:**

Thirty-six Chinese participants were enrolled, with 32 completing the study. The incidence of adverse events was similar between the rHA and HSA groups (44.4% vs. 44.4%, *p* > 0.05). Serum albumin concentration increases were comparable between groups during treatment and follow-up. While most participants experienced weight and abdominal circumference decreases, no significant dose effect was observed (*p* > 0.05). No anti-drug antibodies were detected.

**Conclusion:**

In this study, rHA demonstrated similar safety and PK/PD to HSA in cirrhotic patients with ascites. rHA was well-tolerated, supporting the need to evaluate its safety and efficacy in a phase II clinical study.

**Supplementary Information:**

The online version contains supplementary material available at 10.1007/s12072-025-10871-x.

## Introduction

Ascites is a common and severe complication of cirrhosis, often indicating hepatic decompensation and disease progression, with significant implications for prognosis [1]. Although diuretics and ultrasound-guided paracentesis are widely used to manage ascites [2], treatment remains challenging. A key pathophysiological feature of ascites in cirrhosis is hypoalbuminemia, driven by impaired albumin synthesis, accelerated catabolism, and altered plasma volume due to liver dysfunction [3]. Long-term albumin administration in patients with decompensated cirrhosis improves survival, reduces complications, and aids in ascites control [4].

Human serum albumin (HSA), a 67 kDa, non-glycated protein consisting of 585 amino acids [5], is the most abundant protein in human plasma. It plays a vital role in maintaining plasma colloid osmotic pressure (PCOP) [6] and in binding, transporting, and detoxifying various endogenous and exogenous substances [7, 8]. For decades, HSA has been widely used to treat ascites secondary to liver cirrhosis [9]. However, despite rigorous manufacturing controls, plasma-derived HSA carries a risk of contamination by pathogens such as HIV, hepatitis B and C viruses, cytomegalovirus, and prions [10, 11]. In addition, HSA production depends on plasma, a finite resource, limiting its scalability and accessibility. These challenges highlight the need for safer, more sustainable alternatives.

Recombinant human serum albumin (rHA) addresses both of those concerns. Moreover, rHA and HSA exhibit comparable safety and tolerability. However, despite large-scale production through genetic engineering, concerns persist regarding the potential presence of host-cell proteins (HCPs) and altered glycosylation patterns, which may provoke immune responses [12, 13]. These issues raise questions about the safety of rHA, especially in diseased populations.

Therefore, this study aims to evaluate the safety, tolerability, and pharmacokinetics/pharmacodynamics (PK/PD) of rHA compared to HSA in this patient population. It will inform dose selection and treatment strategies for future clinical trials.

## Materials and methods

### Clinical study design and participants

It was a randomized, open-label, phase Ib trial (ClinicalTrials.gov ID: NCT04701697) designed to evaluate the safety, tolerability, and PK/PD of rHA in patients with cirrhotic ascites. The study was conducted between December 2019 and September 2020 at three medical centers in China: The First Hospital of Jilin University, Tonghua Central Hospital, and the Affiliated Hospital of Yanbian University. Participants included adults diagnosed with ascites resulting from grade 1 or 2 decompensated cirrhosis.

The inclusion criteria were: adults aged 18–75 years, with a weight ≥ 45 kg and a BMI between 18.5 and 28.0 kg/m^2^, diagnosed with ascites due to grade 1 or 2 decompensated cirrhosis (according to EASL guidelines), and a serum albumin concentration < 30 g/L. Exclusion criteria included: a history of allergic reactions to Escherichia coli, yeast, Chinese hamster ovary cell products, human albumin, or other blood products; a diagnosis of hepatorenal syndrome or hepatic encephalopathy (grade III or higher); serum creatinine > 2 × ULN or a > 50% increase during screening; organ transplant recipients; pregnant or breastfeeding women, or women unwilling to use contraception; serious underlying diseases (e.g., severe infections or cardiovascular conditions); participation in other clinical trials or use of investigational products within 3 months; significant laboratory abnormalities (e.g., PLT < 30 × 10⁹/L, HGB < 70 g/L, ALT/AST > 5 × ULN, TBIL > 3 × ULN); and any other conditions deemed inappropriate for participation by the investigator.

### Sample size

According to the “Technical Guidelines for Clinical Pharmacokinetic Studies of Chemical Drugs (2005)”, each dosage group requires 8–12 participants. Since this trial involves a biologic macromolecule, and based on the guidelines, each dosage group includes nine participants, with an additional three participants in each group assigned as positive controls using human albumin. Therefore, a total of 36 participants were included in this study.

### Randomization, masking, and research group

Randomization lists for the study were generated using SAS V9.4 software. Participants were randomly assigned in a 9:3 ratio to receive either rHA or HSA. Participants were divided into three dose groups (10 g/day, 20 g/day, or 30 g/day). The dose escalation in this trial starts at 10 g/day and progresses sequentially to 20 g/day and 30 g/day. Dose escalation requires the completion of tolerance observations in at least 6 participants per group before advancing to the next dose level. For the 10 g/day group, a minimum observation period of 7 days is required, while for the 20 g/day and 30 g/day groups, a minimum observation period of 14 days is necessary. If the tolerance of the participants cannot be determined within the specified observation period, the observation period may be extended. The criteria for terminating dose escalation included: (a) more than five participants experiencing grade 2 or higher adverse events (AEs) as per CTCAE version 5.0; (b) more than three participants developing grade 3 or higher AEs; (c) one participant experiencing a serious adverse event (SAE) related to the investigational drug; or (d) any other safety concerns warranting early termination, as determined by the investigator or sponsor. Safety data were reviewed at each dose level, beginning with the lowest dose, and the next higher dose was initiated only if the safety data from the lower dose were favorable. The maximum treatment duration for each group was 14 days or until plasma albumin levels reached 35 g/L. The study was approved by the First Hospital of Jilin University Committee, with signed informed consent obtained before participation.

### Drug administration

The experimental product was 20% rHA (10 g/vial, 50 mL), manufactured by Tonghua Anrate Biopharmaceuticals Co., Ltd. (Lot Numbers: ErHAQ20190505, ErHAQ20190629, ErHAQ20191025, ErHAQ20200331, ErHAQ20200402). The positive control was 20% HAS (10 g/vial, 50 mL), manufactured by Shanghai Raas Blood Products Co., Ltd. (Lot Number: 201806A023) and supplied by Tonghua Anrate Biopharmaceuticals Co., Ltd. Both HSA and rHA were administered intravenously at a rate of 50 mL/h.

### Study outcomes

#### Primary outcome

The primary outcome of this study was the safety of rHA injection, which was assessed through the incidence and severity of AEs related to rHA treatment. Safety assessments included monitoring AEs, clinical laboratory test abnormalities, vital signs, physical examinations, and 12-lead electrocardiography. AEs were categorized by frequency and severity using the National Cancer Institute Common Terminology Criteria for Adverse Events, version 5.0 (CTCAE v5.0). Drug–AE causality was classified into five categories: unrelated, unlikely, possibly, probably, or definitely related. Tolerability was measured by the number of participants who met the dose escalation termination criteria.

#### Secondary outcome

The secondary outcomes included: (a) major efficacy character: change in albumin concentration before and after treatment; (b) efficacy: change in ascites regression rate, ascites resolution time, abdominal circumference and weight before and after treatment; (c) PD and PK characteristics of rHA following both single-dose and repeated increasing-dose administrations; (d) Immunogenicity: screening for anti-rHA antibodies was performed using a bridging electrochemiluminescence immunoassay (Bridging-ECLIA) on blood samples collected both before and 28 days after the first infusion. Participants who provided at least one plasma specimen were included in the PK and PD analysis set. Serum albumin levels were used as PK parameters, and PCOP, which is defined as the osmotic pressure exerted by proteins and other macromolecules in plasma, was used as a PK index. PCOP was measured using a BMT923 device (BMT MESSTECHNIK GMBH, Berlin, Germany). The BMT 923 uses a semipermeable membrane to measure the pressure difference between the sample and a reference solution.

For the PK analysis, blood samples were collected on Day 1 at 0 h (pre-infusion), at infusion termination (+ 5 min), and 24 h (± 30 min) post-infusion. Subsequent samples for Valley concentration were taken at 0 h before infusion each day following Day 1 and at infusion termination (+ 5 min) on the last day. Additional samples were collected at 24 h (± 30 min), 96 h (± 24 h), 168 h (± 24 h), 336 h (± 24 h), and 672 h (± 24 h) after the last infusion. For the PD analysis, blood samples were taken at 0 h (pre-infusion) on each infusion day and at 2, 5, 8, 15, 22, and 29 days post-final infusion.

The Child–Pugh classification was a scoring system used to evaluate liver function in patients with cirrhosis, categorized into three grades: Grade A (5–6 points) indicated relatively preserved liver function, Grade B (7–9 points) indicated moderate impairment, and Grade C (10–15 points) indicated severe liver dysfunction [14].

### Statistical analysis

Descriptive statistics were used to analyze demographic parameters. Serum albumin concentrations were summarized and compared across dose groups using descriptive statistics. The mean values of PCOP were also described using descriptive statistics. All analyses were performed using SAS® version 9.4 (SAS Institute Inc. NC, USA). *p* values less than 0.05 were considered statistically significant.

## Results

### Study participants

**Fig. 1:**
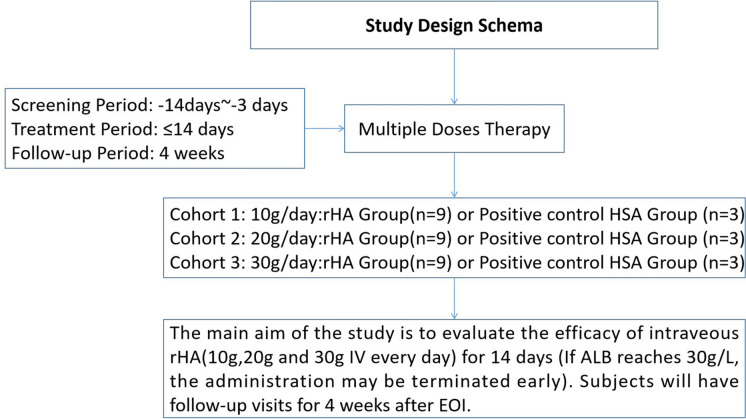
Flow chart of the study

A total of 36 patients with liver cirrhosis and ascites were finally considered for this study, including 27 in the rHA group and 9 in the HAS (positive-control) group. Of the participants, six were diagnosed with hepatocellular carcinoma (HCC). Two were at BCLC stage A, asymptomatic, with small lesions and preserved liver function (Child–Pugh B), and were included following comprehensive evaluation. Four had stable disease after curative treatment (e.g., surgery or radiofrequency ablation). To ensure homogeneity, patients with BCLC stage B or higher HCC, other malignancies (e.g., lung cancer), or urine protein levels ≥ 2+ were excluded based on investigator judgment. Four participants withdrew (three from the rHA group, one from the HSA group). Ultimately, 32 patients completed the study. The study flowchart is presented in Fig. 1.

The demographic characteristics of the participants are summarized in Table 1. The average age was 55.8 ± 7.59 years, the average height was 166.59 ± 8.02 cm, and the average weight was 65.28 ± 10.73 kg at baseline. The average body mass index (BMI) was 23.42 ± 2.76 kg/m^2^. No significant differences in age or BMI were observed between the HSA and rHA groups. Child–Pugh B was seen in 17 participants (63.0%) in the rHA group and six (66.7%) in the HSA group, while Child–Pugh C was observed in 10 (37.0%) and three (33.3%) participants in the rHA and HSA groups, respectively. There were 15 participants with hepatitis B, three with hepatitis C, 13 with gallstones, seven with hypertension, six with diabetes, six with primary liver cancer, four with urinary tract infection, six with liver cysts, two with renal cysts, five with peptic ulcer, and one with psoriasis.

**Table 1:** Baseline characteristics of the included participants

	10 g		20 g		30 g		Total		Total (*N* = 36)
	rHA (*N* = 9)	HSA (*N* = 3)	rHA (*N* = 9)	HSA (*N* = 3)	rHA (*N* = 9)	HSA (*N* = 3)	rHA (*N* = 27)	HSA (*N* = 9)	
Age (years)	56.7 ± 7.42	49.3 ± 10.21	53.8 ± 8.00	55.0 ± 7.81	59.3 ± 6.02	55.7 ± 9.02	56.6 ± 7.29	53.3 ± 8.41	55.8 ± 7.59
Gender [*n* (%)]									
Male	7 (77.8)	3 (100.0)	4 (44.4)	2 (66.7)	8 (88.9)	2 (66.7)	19 (70.4)	7 (77.8)	26 (72.2)
Female	2 (22.2)	0 (0.0)	5 (55.6)	1 (33.3)	1 (11.1)	1 (33.3)	8 (29.6)	2 (22.2)	10 (27.8)
Nationality [*n* (%)]									
Han nationality	9 (100.0)	3 (100.0)	7 (77.8)	3 (100.0)	8 (88.9)	3 (100.0)	24 (88.9)	9 (100.0)	33 (91.7)
Others	0 (0.0)	0 (0.0)	2 (22.2)	0 (0.0)	1 (11.1)	0 (0.0)	3 (11.1)	0 (0.0)	3 (8.3)
Height (cm)	167.98 ± 9.275	166.73 ± 3.035	160.76 ± 8.802	166.33 ± 1.155	169.34 ± 6.490	171.83 ± 7.422	166.03 ± 8.832	168.30 ± 4.844	166.59 ± 8.019
Weight (kg)	66.82 ± 13.600	64.20 ± 6.227	55.60 ± 7.380	71.40 ± 4.513	70.73 ± 9.405	68.30 ± 2.095	64.39 ± 11.984	67.97 ± 5.066	65.28 ± 10.725
BMI (kg/m^2^)	23.51 ± 3.135	23.07 ± 2.285	21.51 ± 2.252	25.77 ± 1.274	24.62 ± 2.675	23.27 ± 2.658	23.21 ± 2.917	24.03 ± 2.275	23.42 ± 2.763
MAP (mmHg)	89.70 ± 11.100	89.77 ± 6.801	87.56 ± 9.439	81.33 ± 14.724	88.67 ± 9.690	97.78 ± 6.882	88.31 ± 9.718	90.63 ± 11.328	89.39 ± 9.975
Heart rate (bpm)	80.2 ± 8.67	76.3 ± 4.04	79.4 ± 8.16	78.3 ± 9.29	81.3 ± 8.72	79.3 ± 3.06	80.3 ± 8.22	78.0 ± 5.45	79.8 ± 7.62
Ca (mmol/l)	2.16 ± 0.152	2.21 ± 0.142	2.17 ± 0.135	2.21 ± 0.160	2.19 ± 0.140	2.13 ± 0.061	2.17 ± 0.142	2.19 ± 0.134	2.18 ± 0.142
K (mmol/l)	4.58 ± 0.580	4.30 ± 0.917	4.57 ± 0.676	4.53 ± 0.416	4.51 ± 0.573	4.33 ± 0.971	4.56 ± 0.585	4.39 ± 0.708	4.51 ± 0.614
Na (mmol/l)	137.67 ± 3.808	136.33 ± 4.509	137.33 ± 3.536	138.67 ± 4.502	138.11 ± 3.257	135.67 ± 4.726	137.70 ± 3.417	136.89 ± 4.197	137.50 ± 3.512
Cr (µmol/l)	73.62 ± 16.808	71.43 ± 13.578	71.23 ± 15.285	65.77 ± 9.051	67.73 ± 17.656	73.17 ± 11.392	70.86 ± 16.150	70.12 ± 10.500	70.68 ± 14.802
eGFR (mL/min/1.73 m^2^)	102.61 ± 9.149	108.16 ± 6.897	105.59 ± 12.352	106.83 ± 8.979	103.29 ± 15.185	103.45 ± 7.948	103.83 ± 13.192	106.15 ± 10.457	104.97 ± 11.283

*BMI* body-mass index, *SD* standard deviation, *n* number of participants, *rHA* recombinant human serum albumin, *HSA* human serum albumin, *MAP* mean arterial pressure, *Cr* creatinine, *eGFR* estimated glomerular filtration rate

### Safety

The rHA treatment was well tolerated in patients with ascites due to cirrhosis. All three planned doses were tested, as the dose escalation termination criteria were unmet. The safety results are summarized in Tables 2, S1, and S2.

**Table 2:** Adverse events after administration of rHA or HSA

	10 g				20 g				30 g				Total				Total	
	rHA		HSA		rHA		HSA		rHA		HSA		rHA		HSA			
	(*N* = 9)		(*N* = 3)		(*N* = 9)		(*N* = 3)		(*N* = 9)		(*N* = 3)		(*N* = 27)		(*N* = 9)		(*N* = 36)	
	*n* (%)	*m*	*n* (%)	*m*	*n* (%)	*m*	*n* (%)	*m*	*n* (%)	*m*	*n* (%)	*m*	*n* (%)	*m*	*n* (%)	*m*	*n* (%)	*m*
TEAE	6 (66.7)	16	2 (66.7)	3	4 (44.4)	9	0 (0.0)	0	2 (22.2)	3	2 (66.7)	4	12 (44.4)	28	4 (44.4)	7	16 (44.4)	35
Research drug-related TEAE	0 (0.0)	0	0 (0.0)	0	1 (11.1)	1	0 (0.0)	0	0 (0.0)	0	0 (0.0)	0	1 (3.7)	1	0 (0.0)	0	1 (2.8)	1
TEAE of grade 3^1^ or above	1 (11.1)	2	2 (66.7)	2	1 (11.1)	2	0 (0.0)	0	2 (22.2)	2	1 (33.3)	1	4 (14.8)	6	3 (33.3)	3	7 (19.4)	9
TEAE leading to reduced dose	0 (0.0)	0	0 (0.0)	0	0 (0.0)	0	0 (0.0)	0	0 (0.0)	0	0 (0.0)	0	0 (0.0)	0	0 (0.0)	0	0 (0.0)	0
TEAE leading to drug suspension	0 (0.0)	0	0 (0.0)	0	1 (11.1)	1	0 (0.0)	0	0 (0.0)	0	0 (0.0)	0	1(3.7)	1	0 (0.0)	0	1 (2.8)	1
TEAE leading to drug termination	0 (0.0)	0	0 (0.0)	0	0 (0.0)	0	0 (0.0)	0	0 (0.0)	0	0 (0.0)	0	0 (0.0)	0	0 (0.0)	0	0 (0.0)	0
TEAE leading to participants withdrawal from the study	0 (0.0)	0	0 (0.0)	0	0 (0.0)	0	0 (0.0)	0	0 (0.0)	0	0 (0.0)	0	0 (0.0)	0	0 (0.0)	0	0 (0.0)	0
SAE	0 (0.0)	0	1 (33.3)	1	1 (11.1)	1	0 (0.0)	0	2 (22.2)	2	0 (0.0)	0	3 (11.1)	3	1 (11.1)	1	4 (11.1)	4
SAE related to drug	0 (0.0)	0	0 (0.0)	0	0 (0.0)	0	0 (0.0)	0	0 (0.0)	0	0 (0.0)	0	0 (0.0)	0	0 (0.0)	0	0 (0.0)	0
SAE leading to death	0 (0.0)	0	0 (0.0)	0	0 (0.0)	0	0 (0.0)	0	0 (0.0)	0	0 (0.0)	0	0 (0.0)	0	0 (0.0)	0	0 (0.0)	0

The Common Terminology Criteria for Adverse Events (CTCAE 5.0) was used to grade adverse events (grade 1–5)

*rHA* recombinant human serum albumin, *HAS* human serum albumin, *TEAE* treatment-emergent adverse events, *N* number of participants analyzed, *n* number of participants with adverse events, *m* number of adverse even

**Fig. 2:**
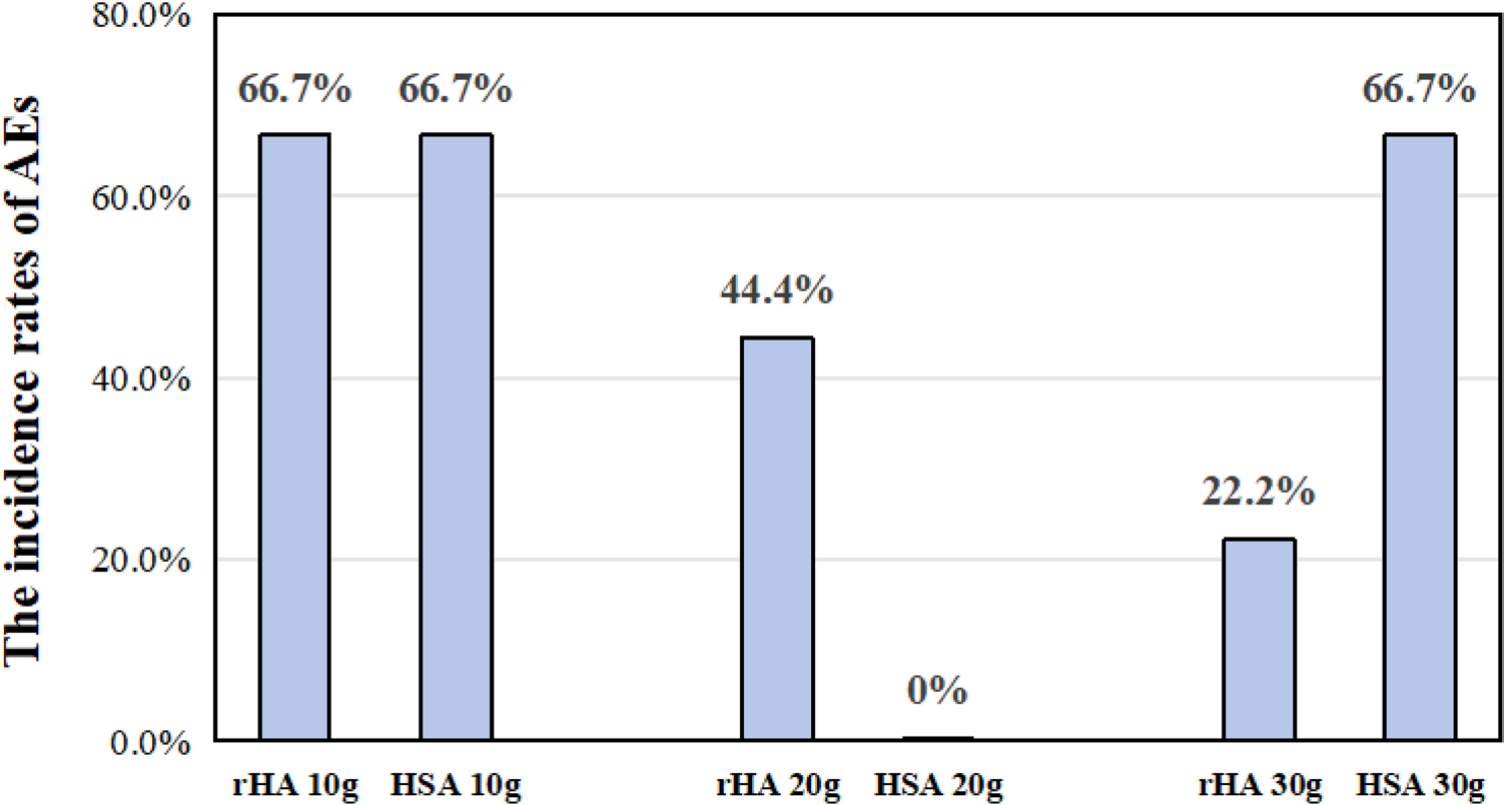
Comparison of the adverse events according to drug doses

The overall incidence of AEs was 44.4% (16/36), including 12 cases (44.4%) in the rHA group and 4 cases (44.4%) in the HSA group. In the rHA group, AEs occurred in 6 participants (66.7%) at 10 g/day, 4 at 20 g/day (44.4%), and 2 at 30 g/day (22.2%). In the HSA group, AEs occurred in 2 participants at 10 g/day (66.7%), 0 at 20 g/day (0%), and 2 at 30 g/day (66.7%) (Fig. 2). The incidence of AEs was similar between the two groups but did not correlate with the dose.

Cough was observed only in one patient in the HAS group (11.1%). The symptoms fully resolved (no sequelae) after treatment with oseltamivir phosphate, Lianhua Qingwen granules, Qiangli Pipa capsules, and nystatin). The investigator determined it was unrelated to the study drug. Peripheral edema was observed in one patient in the rHA group (3.7%). Bilateral lower limb edema was seen in one case in the rHA group (3.7%). No treatment was administered, and the patient experienced relief or complete recovery (no sequelae). There were no cases of dyspnea or muscle pain.

One participant (3.7%) in the 20 g/day rHA group developed a fever, and was determined to be due to ascites infection, which was not related to treatment. According to the Common Terminology Criteria for Adverse Events (CTCAE) version 5.0, grade 3 AEs occurred in one participant each in the 10 g/day rHA group, 20 g/day rHA group, and 30 g/day HSA group. Two cases of grade 3 AEs were observed in the 10 g/day HSA and 30 g/day rHA groups. The remaining AEs were grade 1 or grade 2. Laboratory test abnormalities, ECG changes, vital sign fluctuations, physical examination findings, and thyroid function test abnormalities were observed only in the rHA group, with a low incidence. There was no correlation between treatment-emergent AEs and dose. Three participants, one from each dose group, had clinically significant abnormal laboratory findings: two with decreased white blood cell counts and one with low platelet levels. Two participants, one in the 10 g/day group and one in the 20 g/day group, had abnormal ECG findings, which were recorded as AEs. No significant trends in vital signs were observed during the study. One participant in the 30 g/day group (11.1%) experienced bilateral lower-extremity edema, and one in the 20 g/day group (11.1%) developed subclinical hypothyroidism.

Four SAEs were reported: bile duct stones in the 10 g/day HSA group, nephrotic syndrome in the 20 g/day rHA group, gastrointestinal bleeding in the 30 g/day rHA group, and incarcerated inguinal hernia in the 30 g/day rHA group. The participant with bile duct stones had a clear diagnosis before enrollment and required surgical treatment, which was postponed due to poor liver function. Surgery was performed on the 8th day after the last dose of HSA. The participant with nephrotic syndrome had a history of positive urinary protein and was hospitalized for systemic treatment on the 11th day after the last dose of rHA. These SAEs were considered unrelated to the study drug, given the participants’ medical histories. The participant with gastrointestinal bleeding SAE experienced melena 12 days after the last dose of rHA. Gastroscopy revealed severe esophageal varices due to portal hypertension caused by liver cirrhosis, and esophageal variceal ligation was performed. On the 12th day after the last dose of rHA, a participant presented with a hard mass and pain in the right inguinal region. Doppler ultrasound confirmed a right incarcerated inguinal hernia, and surgery was performed on the same day. Both of these SAEs were considered unrelated to the study drug and instead attributed to the progression of the underlying disease.

Among the four SAE cases, the bile duct stones, gastrointestinal bleeding, and right incarcerated inguinal hernia were considered “completely recovered” by the end of the study, while nephrotic syndrome was considered “in remission.”

### PK and PD

**Fig. 3:**
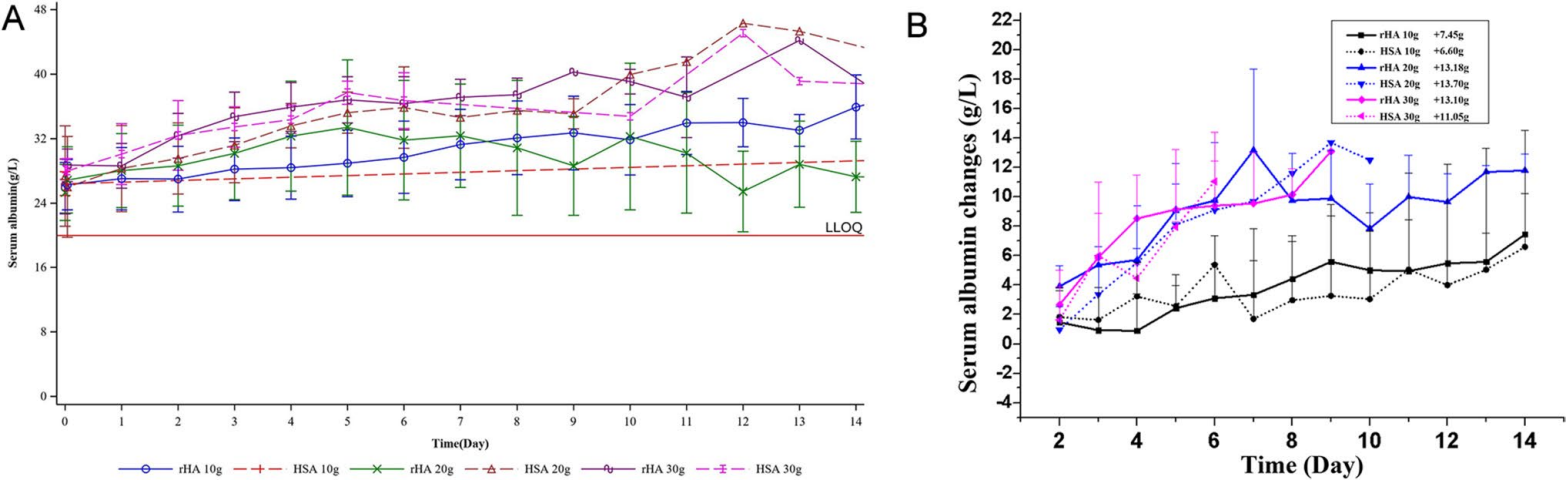
Pharmacokinetics profiles of rHA and HSA in patients with ascites due to cirrhosis. **a** Mean serum albumin concentration (g/L). **b** Changes in mean serum albumin concentration

Data collected from 35 participants were included in the PK analyses (Fig. 3 and Figure S1). The trends in serum albumin concentration changes were similar between the rHA and HSA groups. Both rHA and HSA treatments led to dose-dependent increases in serum albumin concentrations during the treatment period. During the follow-up period, serum albumin concentrations declined but remained higher than baseline levels 29 days after the last infusion. The rate of serum albumin increase was dose-dependent, with the 20 g/day and 30 g/day groups showing significantly greater increases compared to the 10 g/day group. Maximum serum albumin levels achieved by the end of treatment were similar across all dose groups despite a longer treatment duration for the 10 g/day group.

**Fig. 4:**
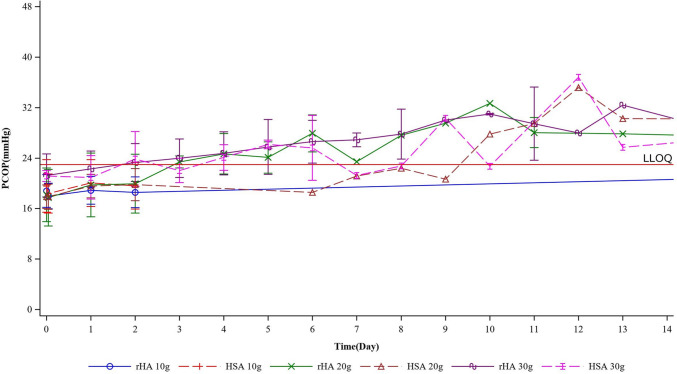
Pharmacodynamic profiles of rHA and HSA in patients with ascites due to cirrhosis. Mean PCOP (mmHg) is shown for each group

As shown in Fig. 4 and Figure S2, the rHA and HSA treatments resulted in similar improvements in PCOP. Mean PCOP significantly increased compared to baseline by day 2 of treatment with 20 g/day or 30 g/day of either rHA or HSA, with further increases observed by the last dose. These improvements in mean PCOP were maintained until day 29 after the last infusion, although no statistically significant differences were observed between the groups. The largest improvements in PCOP were observed in the 20 g/day groups on day 5 after the last dose, with gradual decreases thereafter. The rate of increase in mean PCOP was greater at higher doses for both rHA and HSA, and this rate also correlated with the dose level. Similar values were seen in the 20 g/day and 30 g/day groups, which was likely due to the shorter treatment duration in the latter. Changes in PCOP mirrored serum albumin trends throughout the study.

As shown in Table 3, the time required to reach a serum albumin level of 35 g/L was dose-dependent in both groups, with higher doses resulting in shorter times to reach the target level.

**Table 3:** Time for serum albumin concentration to reach 35 g/L

	10 g		20 g		30 g	
	rHA (*N* = 9)	HSA (*N* = 3)	rHA (*N* = 9)	HSA (*N* = 3)	rHA (*N* = 9)	HSA (*N* = 3)
*n* (missing)	6 (3)	2 (1)	7 (2)	3 (0)	9 (0)	3 (0)
Mean (SD)	14.0 (4.60)	10.5 (3.54)	7.4 (5.29)	6.3 (3.21)	5.3 (2.50)	4.3 (1.15)
Median	12.5	10.5	5.0	5.0	5.0	5.0
Q1, Q3	10, 18	8, 13	4, 10	4, 10	4, 5	3, 5
Min, Max	10, 21	8, 13	3, 18	4, 10	3, 10	3, 5

### Efficacy

After the treatment, the mean changes in abdominal circumference were as follows: − 0.79 cm, − 3.66 cm, and − 2.03 cm for the 10 g/day, 20 g/day, and 30 g/day rHA groups, respectively, and − 6.33 cm, − 4.63 cm, and − 2.83 cm for the corresponding HSA groups (Table S3). The abdominal circumference changes in the 10 g/day, 20 g/day, and 30 g/day rHA groups all showed a downward trend compared to baseline, though no significant dose effect was observed. Notably, the 20 g/day and 30 g/day rHA groups demonstrated significantly better results than the 10 g/day group.

The mean weight changes were − 1.28 kg, − 3.55 kg, and − 0.88 kg for the 10 g/day, 20 g/day, and 30 g/day rHA groups, respectively, and − 1.23 kg, − 1.60 kg, and − 0.43 kg for the corresponding HSA groups (Table S4). Although weight decreased in most participants, no significant dose effect was observed. The 20 g/day group experienced the greatest weight loss, possibly due to sample size imbalances and individual variations.

### Immunology

Anti-rHA antibodies were measured before and 28 days after the first infusion, with no anti-drug antibodies detected.

## Discussion

This randomized, open-label, phase Ib trial is the first to evaluate the safety, tolerability, PK/PD, and efficacy of rHA in Chinese patients with ascites due to cirrhosis. The study found that rHA was well tolerated, with no treatment-related AEs and no evidence of immunologic responses. In addition, rHA demonstrated a favorable PK/PD profile, suggesting that rHA holds great promise as an alternative to plasma-derived HSA in clinical applications. Although the sample size was small, the results are encouraging and provide preliminary evidence supporting the safety and efficacy of rHA. These findings set the stage for further studies with larger cohorts and longer follow-ups to confirm the efficacy and long-term safety of rHA in cirrhotic patients with ascites.

Safety is a primary concern when introducing rHA for clinical use, particularly regarding potential allergic responses. In this study, rHA was well tolerated by the participants, with no serious allergic AEs or anaphylaxis reported. The incidence and nature of drug-related AEs were minimal, supporting the safety profile of rHA in this patient population. Four SAEs were reported; however, these were deemed unrelated to the study drug: two cases of bile duct stones and nephrotic syndrome, both with clear pre-existing histories; one case of gastrointestinal bleeding, which was linked to portal hypertension; and one case of right incarcerated inguinal hernia, which was also unrelated to the study drug. In contrast to HSA, which has been associated with occasional hypersensitivity reactions and other complications [15], rHA demonstrated a more favorable safety profile. It is also important to note that the FDA has highlighted a risk of fatal pulmonary edema with excessive albumin use [16], especially in patients treated with terlipressin. However, in this study, we closely monitored vital signs, respiratory status, and the occurrence of AEs, with no respiratory distress or similar AEs observed. In addition, the study included monitoring of other laboratory indicators, all of which remained stable throughout the treatment period. The dose–response relationship was also explored, with no clear evidence of increasing AEs at higher doses. It further suggests that rHA could be a safe alternative for cirrhotic patients with ascites.

Recombinant proteins for biopharmaceutical applications are produced in various expression systems, but HCPs may serve as contaminants that can impact safety. Although HCPs are typically present in low quantities, significant efforts and costs are invested to remove them [17]. In this study, anti-rHA antibodies were measured before and 28 days after the first infusion, and no anti-drug antibodies were detected. It indicates that HCPs were effectively removed during the manufacturing process, significantly enhancing the safety profile of rHA.Future studies with larger sample sizes and extended follow-up periods are necessary to confirm these findings and explore the long-term safety of rHA in this clinical context.

Both rHA and HSA groups exhibited similar trends in serum albumin concentration. Serum albumin levels increased during treatment, with the rate and magnitude of increase proportional to the dose of rHA or HSA. The 20 g/day and 30 g/day groups showed comparable increases, while the 10 g/day group showed slower and smaller increases. Although serum albumin levels declined during the follow-up period, they remained elevated compared to baseline at day 29 after the last infusion. A limitation of this study is that we could not distinguish rHA from endogenous albumin, preventing the calculation of rHA’s half-life. A previous study has shown that HSA has a turnover time of approximately 25 days and an elimination half-life of 17.3 days [18]. The results of our study indicate that rHA has a long half-life in the human body and can exert its therapeutic effect stably, even though the exact half-life could not be calculated.

Albumin accounts for 80% of PCOP and is the main regulator of the dynamic water balance between tissues and blood vessels [9]. Our results show that rHA treatment can effectively boost PCOP, with a dose-dependent effect similar to that of HSA. It provides valuable insight into the PD of rHA in cirrhotic patients with ascites, as comparative data on the pharmacodynamic effects of HSA in this context are limited.

In the 30 g/day groups, 55.6% of participants had a history of portal hypertension, and 11.1% had portal thrombosis and cavernous degeneration, which indicates that high doses of albumin were safely administered to these patients. Interestingly, the higher the albumin dose, the less time it took to reach the target serum albumin level of 35 g/L. The average treatment time in the 10 g/day group was 11.9 days, whereas that in the 20 g/day and 30 g/day groups was 7.3 days and 5.4 days, respectively. It suggests that high doses of rHA are safe and effective for rapidly increasing serum albumin levels when clinically necessary.

Our phase 1b study had several inherent limitations to consider when interpreting the results. Firstly, the open-label design introduced potential biases that could have influenced participant outcomes and researcher assessments. The small sample size of the trial further constrained the generalizability of the findings. Due to the lack of statistical power in these comparisons, the exploratory and secondary analyses of this study should be interpreted with caution. In addition, the relatively short follow-up period may not have adequately captured long-term clinical outcomes, highlighting the necessity for further investigations with extended follow-up durations. Long-term safety (including anti-albumin antibodies) could not be assessed, and the patients were not followed up on in the long term. Long-term safety monitoring will be conducted in our future studies. Furthermore, the study’s experimental design precluded analysis of changes in albumin levels, which may have impacted result interpretation. Future research should incorporate such dynamic analyses. Despite these limitations, the preliminary data were promising, underscoring the need for larger-scale, multicenter cohorts and trials with prolonged follow-up in future.

Based on the present study, a Phase II clinical trial was designed to evaluate the dose–response, safety, and immunogenicity of rHA injection in patients with liver cirrhosis and ascites-related hypoalbuminemia, providing a basis for the Phase III clinical trial design. The patients receive an albumin injection 10 g/day once daily for 14 days or an albumin injection 20 g/day once daily for 7 days. Indeed, based on the results of the Phase Ib clinical trial, intravenous infusion of rHA at 10, 20, and 30 g/day was considered safe and well-tolerated in patients with liver cirrhosis and ascites, and most participants achieved the target serum albumin of 35 g/L. Considering the commonly used clinical doses of albumin, the Phase II trial selected 10 and 20 g/day to assess the dose–response effect. According to the Phase Ib clinical trial results, intravenous infusion of albumin injection at 10 g/day for 14 days or 20 g/day for 7 days should allow most patients with liver cirrhosis and ascites to achieve the target serum albumin levels of 35 g/L. Each group (10 and 20 g/day) will include 45 participants. The participants are assigned 2:1 within each dose group to receive either rHA or HSA.

In summary, most participants in the rHA and HSA groups showed improvement, with no significant dose-dependent effects observed. The results suggest that rHA is safe and well tolerated in cirrhotic patients with ascites, demonstrating PK/PD profiles and efficacy comparable to HSA. A large-scale study in cirrhotic patients with ascites is planned to further investigate the safety and efficacy of rHA.

## Supplementary Information

Below is the link to the electronic supplementary material.

Supplementary file1 (TIF 473 KB)

Supplementary file2 (TIF 392 KB)

Supplementary file3 (DOCX 352 KB)

Supplementary file4 (TIF 731 KB)

## Data Availability

All data generated or analyzed during this study are included in this published article.
