# Supplementary figures and images for: A randomized, phase Ib trial of recombinant human serum albumin in cirrhotic patients with ascites
Source: Hepatol Int. 2025 Jul 23;20(1):91–101. doi: 10.1007/s12072-025-10871-x (PMC12923478; doi:10.1007/s12072-025-10871-x)

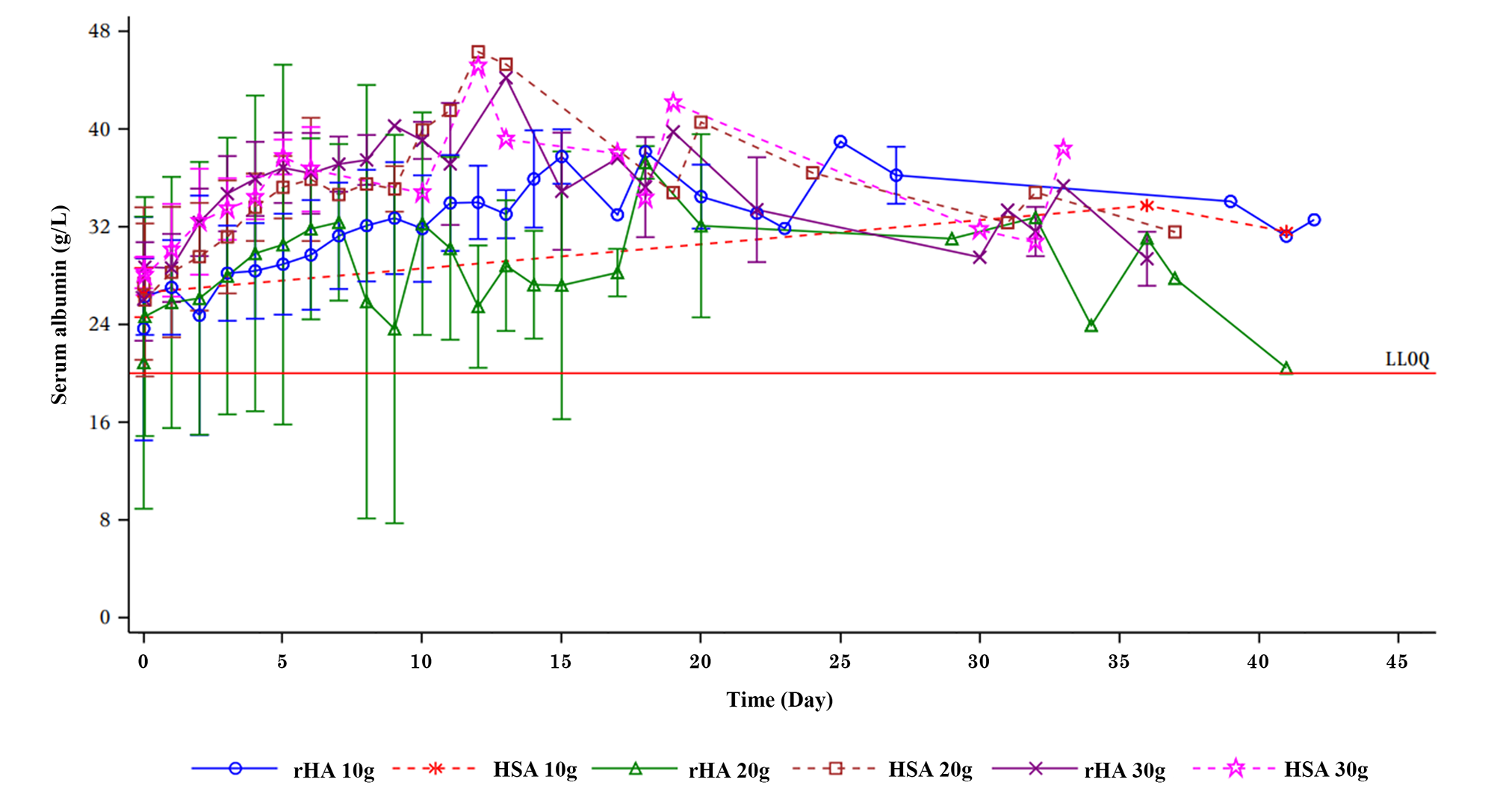

Supplement: Supplementary file 1 — Supplementary file1 (TIF 473 KB) [file 12072_2025_10871_MOESM1_ESM.tif]

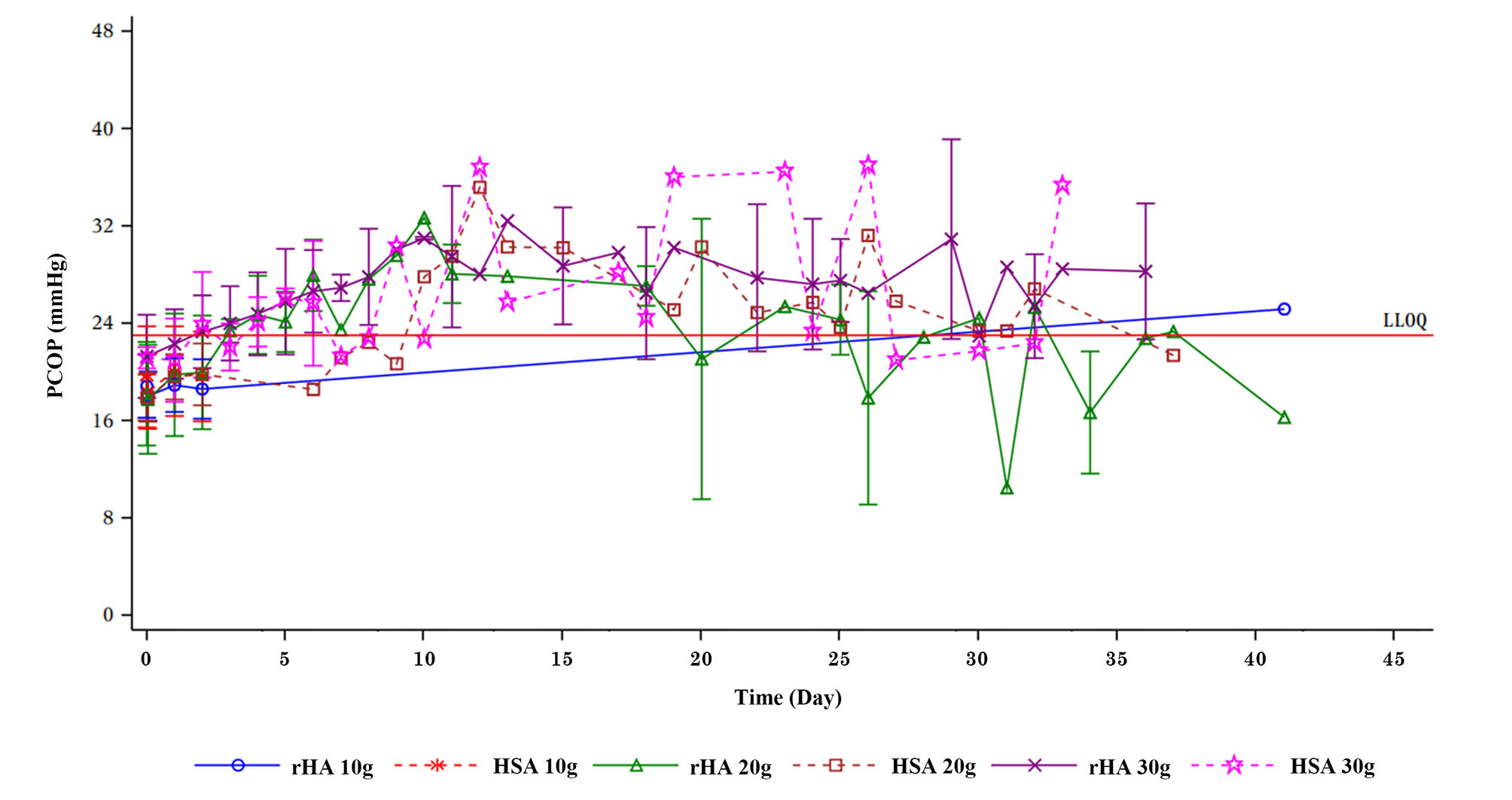

Supplement: Supplementary file 2 — Supplementary file2 (TIF 392 KB) [file 12072_2025_10871_MOESM2_ESM.tif]

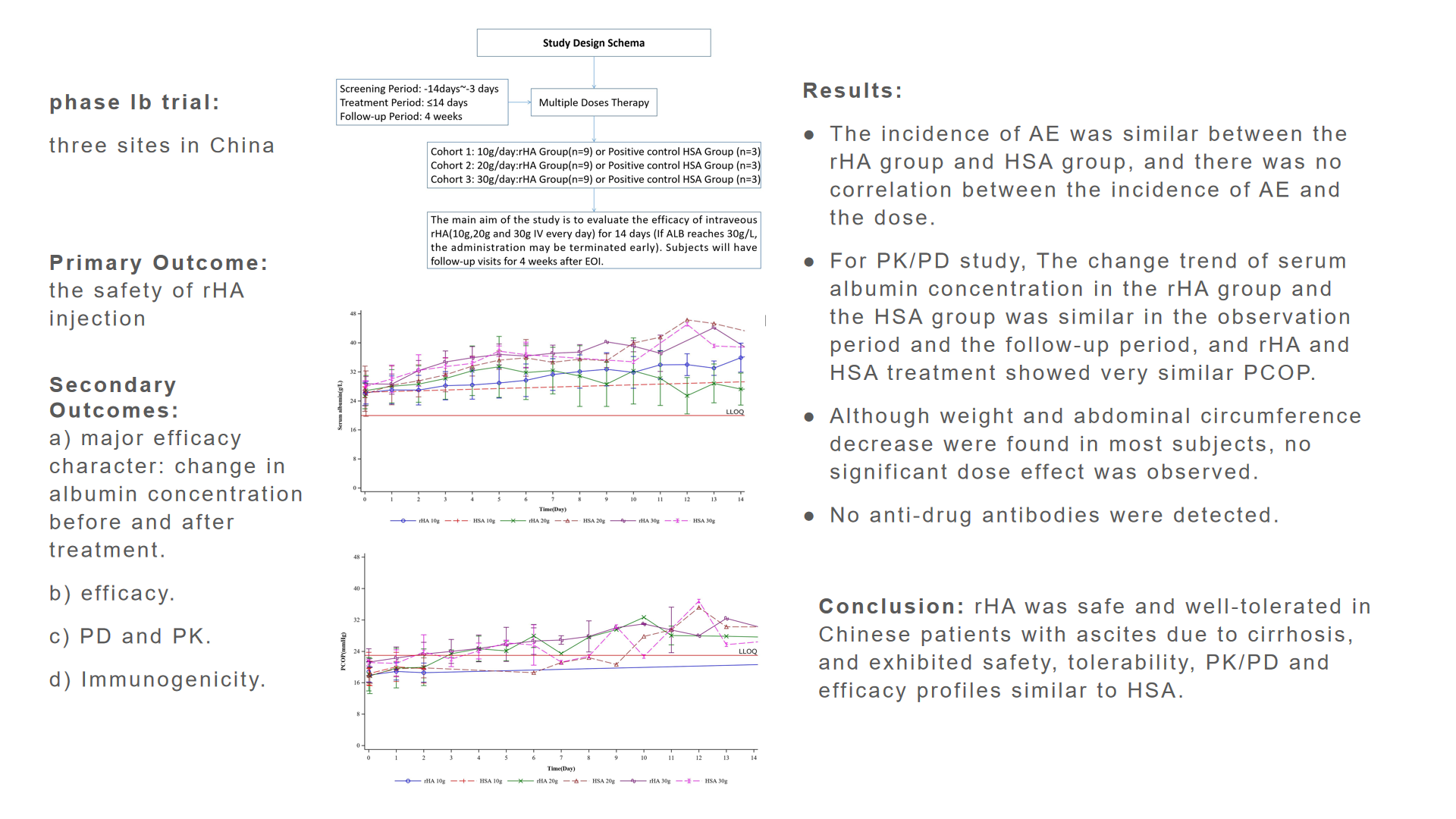

Supplement: Supplementary file 4 — Supplementary file4 (TIF 731 KB) [file 12072_2025_10871_MOESM4_ESM.tif]
