# Supplementary material for: A randomized, phase Ib trial of recombinant human serum albumin in cirrhotic patients with ascites
Source: Hepatol Int. 2025 Jul 23;20(1):91–101. doi: 10.1007/s12072-025-10871-x (PMC12923478; doi:10.1007/s12072-025-10871-x)
Supplement: Supplementary file 3 — Supplementary file3 (DOCX 352 KB) [file 12072_2025_10871_MOESM3_ESM.docx]

**Supplemental Material**

**rHA production information and characterization**

All 585 amino acids of human serum albumin remained unchanged in rHA. Only the nucleotide sequence was optimized based on the codon preference during the construction of the expression vector. Such codon optimization involves threonine 103 and arginine 210:

| Amino acid position | Amino acid | Human codon | Recombinant codon |
| --- | --- | --- | --- |
| 103 | Thr | ACT | ACA |
| 210 | Arg | CGG | CGC |

rHA was produced in bioreactors by the yeast *Pichia pastoris.* The production process of rHA includes the following steps, in that order: primary seed culture, secondary seed culture, tertiary seed culture, main fermentation, collection of fermentation broth, primary centrifugation, heating treatment, centrifugation, microfiltration, ion exchange chromatography, heating treatment II, hydrophobic interaction chromatography, ultrafiltration, macroporous resin separation, ultrafiltration, anion-exchange chromatography, ultrafiltration, affinity chromatography, ultrafiltration, cation-exchange chromatography, anion-exchange chromatography, ultrafiltration, filtration, and bulk preparation. The process impurities in the production of rHA include media components (primarily small-molecule water-soluble substances such as inorganic salts, vitamins, trace elements, soy hydrolysate, yeast extract, macromolecules, methanol, sodium octanoate, heavy metals introduced during the fermentation and production processes and cytochromes, yeast host cell proteins, mannans, and exogenous DNA introduced by *Pichia pastoris*. The main product impurities are polymers, dimers, and degradation fragments produced during the fermentation and purification process. These impurities are removed by purification steps such as microfiltration, ultrafiltration, and chromatography. According to the test results, the residue of these impurities complies with the relevant requirements of pharmacopeia and ICH guidelines, as detailed in the following table:

| Impurities and contaminants | Test results/criteria |
| --- | --- |
| Host cell proteins | ≤ 5 ng/g protein |
| Exogenous DNA residue | ≤ 1 ng/g protein |
| Cytochrome | 0.0002 mol/mol protein |
| Organic solvents | Ethanol, methanol, isopropanol, ethyl acetate, and acetone not detected |
| Antifoam | Not detected |
| Glycerol | Not detected |
| Fatty acids | < 1 mol/mol protein |
| Media components | Not detected |
| Heavy metals | Complies with the PDE specified in the ICH Q3D (R2) *Guideline for Elemental Impurities* |
| Mannan component | < 0.1 mol/mol protein |
| Dimer and polymer | ≤ 2.0% |
| Degradation fragment | Not detected |

Note: A comparative analysis of rHA and human serum albumins from different suppliers was performed using the Thermo Scientific Q Exactive MS system. There were significant differences in the protein glycosylation between Recombinant Human Albumin and human serum albumin. Human serum albumin had a relatively high proportion of glycosylation at S232, with a relative abundance of 17.4%-33.0%, while rHA had a low abundance of mannosylation at S489, not more than 5.0%.

**Table S1**. Incidence of adverse events after administration of rHA or HSA by dose cohort (N=36).

|  | **10 g** | | | | **20 g** | | | | **30 g** | | | | **Total** | | | |  | |
| --- | --- | --- | --- | --- | --- | --- | --- | --- | --- | --- | --- | --- | --- | --- | --- | --- | --- | --- |
|  | **rHA** | | **HSA** | | **rHA** | | **HSA** | | **rHA** | | **HSA** | | **rHA** | | **HSA** | | **Total** | |
|  | **(N = 9)** | | **(N = 3)** | | **(N = 9)** | | **(N = 3)** | | **(N = 9)** | | **(N = 3)** | | **(N = 27)** | | **(N = 9)** | | **(N = 36)** | |
|  | **n (%)** | **m** | **n (%)** | **m** | **n (%)** | **M** | **n (%)** | **m** | **n (%)** | **m** | **n (%)** | **m** | **n (%)** | **m** | **n (%)** | **m** | **n (%)** | **m** |
| TEAE | 6 (66.7) | 16 | 2 (66.7) | 3 | 4 (44.4) | 9 | 0 (0.0) | 0 | 2 (22.2) | 3 | 2 (66.7) | 4 | 12 (44.4) | 28 | 4 (44.4) | 7 | 16 (44.4) | 35 |
| Gastrointestinal diseases | 3 (33.3) | 6 | 1 (33.3) | 1 | 1 (11.1) | 1 | 0 (0.0) | 0 | 2 (22.2) | 2 | 0 (0.0) | 0 | 6 (22.2) | 9 | 1 (11.1) | 1 | 7 (19.4) | 10 |
| Constipation | 2 (22.2) | 2 | 0 (0.0) | 0 | 0 (0.0) | 0 | 0 (0.0) | 0 | 0 (0.0) | 0 | 0 (0.0) | 0 | 2 (7.4) | 2 | 0 (0.0) | 0 | 2 (5.6) | 2 |
| diarrhea | 1 (11.1) | 1 | 0 (0.0) | 0 | 1 (11.1) | 1 | 0 (0.0) | 0 | 0 (0.0) | 0 | 0 (0.0) | 0 | 2 (7.4) | 2 | 0 (0.0) | 0 | 2 (5.6) | 2 |
| Gastrointestinal bleeding | 0 (0.0) | 0 | 1 (33.3) | 1 | 0 (0.0) | 0 | 0 (0.0) | 0 | 1 (11.1) | 1 | 0 (0.0) | 0 | 1 (3.7) | 1 | 1 (11.1) | 1 | 2 (5.6) | 2 |
| Incarcerated inguinal hernia | 0 (0.0) | 0 | 0 (0.0) | 0 | 0 (0.0) | 0 | 0 (0.0) | 0 | 1 (11.1) | 1 | 0 (0.0) | 0 | 1 (3.7) | 1 | 0 (0.0) | 0 | 1 (2.8) | 1 |
| Ascites | 1 (11.1) | 1 | 0 (0.0) | 0 | 0 (0.0) | 0 | 0 (0.0) | 0 | 0 (0.0) | 0 | 0 (0.0) | 0 | 1 (3.7) | 1 | 0 (0.0) | 0 | 1 (2.8) | 1 |
| mouth ulcer | 1 (11.1) | 1 | 0 (0.0) | 0 | 0 (0.0) | 0 | 0 (0.0) | 0 | 0 (0.0) | 0 | 0 (0.0) | 0 | 1 (3.7) | 1 | 0 (0.0) | 0 | 1 (2.8) | 1 |
| Epigastric pain | 1 (11.1) | 1 | 0 (0.0) | 0 | 0 (0.0) | 0 | 0 (0.0) | 0 | 0 (0.0) | 0 | 0 (0.0) | 0 | 1 (3.7) | 1 | 0 (0.0) | 0 | 1 (2.8) | 1 |
|  |  |  |  |  |  |  |  |  |  |  |  |  |  |  |  |  |  |  |
| Systemic diseases and various reactions at the drug administration site | 1 (11.1) | 1 | 0 (0.0) | 0 | 2 (22.2) | 2 | 0 (0.0) | 0 | 1 (11.1) | 1 | 1 (33.3) | 1 | 4 (14.8) | 4 | 1 (11.1) | 1 | 5 (13.9) | 5 |
| Fever | 1 (11.1) | 1 | 0 (0.0) | 0 | 2 (22.2) | 2 | 0 (0.0)) | 0 | 0 (0.0) | 0 | 1 (33.3) | 1 | 3 (11.1) | 3 | 1 (11.1) | 1 | 4 (11.1) | 4 |
| Peripheral edema | 0 (0.0) | 0 | 0 (0.0) | 0 | 0 (0.0) | 0 | 0 (0.0) | 0 | 1 (11.1) | 1 | 0 (0.0) | 0 | 1 (3.7) | 1 | 0 (0.0) | 0 | 1 (2.8) | 1 |
|  |  |  |  |  |  |  |  |  |  |  |  |  |  |  |  |  |  |  |
| Metabolic and nutritional diseases | 1 (11.1) | 1 | 0 (0.0) | 0 | 2 (22.2) | 2 | 0 (0.0) | 0 | 0 (0.0) | 0 | 0 (0.0) | 0 | 3 (11.1) | 3 | 0 (0.0) | 0 | 3 (8.3) | 3 |
| Hypokalemia | 1 (11.1) | 1 | 0 (0.0) | 0 | 1 (11.1) | 1 | 0 (0.0) | 0 | 0 (0.0) | 0 | 0 (0.0) | 0 | 2 (7.4) | 2 | 0 (0.0) | 0 | 2 (5.6) | 2 |
| Hypoglycemia | 0 (0.0) | 0 | 0 (0.0) | 0 | 1 (11.1) | 1 | 0 (0.0) | 0 | 0 (0.0) | 0 | 0 (0.0) | 0 | 1 (3.7) | 1 | 0 (0.0) | 0 | 1 (2.8) | 1 |
|  |  |  |  |  |  |  |  |  |  |  |  |  |  |  |  |  |  |  |
| Tests | 1 (11.1) | 1 | 0 (0.0) | 0 | 1 (11.1) | 1 | 0 (0.0) | 0 | 0 (0.0) | 0 | 1 (33.3) | 1 | 2 (7.4) | 2 | 1 (11.1) | 1 | 3 (8.3) | 3 |
| White blood cell decrease | 1 (11.1) | 1 | 0 (0.0) | 0 | 0 (0.0) | 0 | 0 (0.0) | 0 | 0 (0.0) | 0 | 1 (33.3) | 1 | 1 (3.7) | 1 | 1 (11.1) | 1 | 2 (5.6) | 2 |
| Platelet decrease | 0 (0.0) | 0 | 0 (0.0) | 0 | 1 (11.1) | 1 | 0 (0.0) | 0 | 0 (0.0) | 0 | 0 (0.0) | 0 | 1 (3.7) | 1 | 0 (0.0) | 0 | 1 (2.8) | 1 |
|  |  |  |  |  |  |  |  |  |  |  |  |  |  |  |  |  |  |  |
| Hepatobiliary diseases | 1 (11.1) | 1 | 1 (33.3) | 1 | 0 (0.0) | 0 | 0 (0.0) | 0 | 0 (0.0) | 0 | 0 (0.0) | 0 | 1 (3.7) | 1 | 1 (11.1) | 1 | 2 (5.6) | 2 |
| Cholelithiasis | 0 (0.0) | 0 | 1 (33.3) | 1 | 0 (0.0) | 0 | 0 (0.0) | 0 | 0 (0.0) | 0 | 0 (0.0) | 0 | 0 (0.0) | 0 | 1 (11.1) | 1 | 1 (2.8) | 1 |
| Portal vein thrombosis | 1 (11.1) | 1 | 0 (0.0) | 0 | 0 (0.0) | 0 | 0 (0.0) | 0 | 0 (0.0) | 0 | 0 (0.0) | 0 | 1 (3.7) | 1 | 0 (0.0) | 0 | 1 (2.8) | 1 |
|  |  |  |  |  |  |  |  |  |  |  |  |  |  |  |  |  |  |  |
| Infection and infectious diseases | 1 (11.1) | 2 | 0 (0.0) | 0 | 0 (0.0) | 0 | 0 (0.0) | 0 | 0 (0.0) | 0 | 1 (33.3) | 1 | 1 (3.7) | 2 | 1 (11.1) | 1 | 2 (5.6) | 3 |
| Anal abscess | 1 (11.1) | 2 | 0 (0.0) | 0 | 0 (0.0) | 0 | 0 (0.0) | 0 | 0 (0.0) | 0 | 0 (0.0) | 0 | 1 (3.7) | 2 | 0 (0.0) | 0 | 1 (2.8) | 2 |
| Fungal infection | 0 (0.0) | 0 | 0 (0.0) | 0 | 0 (0.0) | 0 | 0 (0.0) | 0 | 0 (0.0) | 0 | 1 (33.3) | 1 | 0 (0.0) | 0 | 1 (11.1) | 1 | 1 (2.8) | 1 |
|  |  |  |  |  |  |  |  |  |  |  |  |  |  |  |  |  |  |  |
| Respiratory, thoracic and mediastinal diseases | 1 (11.1) | 2 | 0 (0.0) | 0 | 0 (0.0) | 0 | 0 (0.0) | 0 | 0 (0.0) | 0 | 1 (33.3) | 1 | 1 (3.7) | 2 | 1 (11.1) | 1 | 2 (5.6) | 3 |
| Cough | 0 (0.0) | 0 | 0 (0.0) | 0 | 0 (0.0) | 0 | 0 (0.0) | 0 | 0 (0.0) | 0 | 1 (33.3) | 1 | 0 (0.0) | 0 | 1 (11.1) | 1 | 1 (2.8) | 1 |
| Oropharyngeal pain | 1 (11.1) | 1 | 0 (0.0) | 0 | 0 (0.0) | 0 | 0 (0.0) | 0 | 0 (0.0) | 0 | 0 (0.0) | 0 | 1 (3.7) | 1 | 0 (0.0) | 0 | 1 (2.8) | 1 |
| Erythema of pharynx | 1 (11.1) | 1 | 0 (0.0) | 0 | 0 (0.0) | 0 | 0 (0.0) | 0 | 0 (0.0) | 0 | 0 (0.0) | 0 | 1 (3.7) | 1 | 0 (0.0) | 0 | 1 (2.8) | 1 |
|  |  |  |  |  |  |  |  |  |  |  |  |  |  |  |  |  |  |  |
| Kidney and urinary system diseases | 1 (11.1) | 1 | 0 (0.0) | 0 | 1 (11.1) | 1 | 0 (0.0) | 0 | 0 (0.0) | 0 | 0 (0.0) | 0 | 2 (7.4) | 2 | 0 (0.0) | 0 | 2 (5.6) | 2 |
| Oliguria | 1 (11.1) | 1 | 0 (0.0) | 0 | 0 (0.0) | 0 | 0 (0.0) | 0 | 0 (0.0) | 0 | 0 (0.0) | 0 | 1 (3.7) | 1 | 0 (0.0) | 0 | 1 (2.8) | 1 |
| Nephrotic syndrome | 0 (0.0) | 0 | 0 (0.0) | 0 | 1 (11.1) | 1 | 0 (0.0) | 0 | 0 (0.0) | 0 | 0 (0.0) | 0 | 1 (3.7) | 1 | 0 (0.0) | 0 | 1 (2.8) | 1 |
|  |  |  |  |  |  |  |  |  |  |  |  |  |  |  |  |  |  |  |
| Blood and lymphatic system diseases | 0 (0.0) | 0 | 1 (33.3) | 1 | 1 (11.1) | 1 | 0 (0.0) | 0 | 0 (0.0) | 0 | 0 (0.0) | 0 | 1 (3.7) | 1 | 1 (11.1) | 1 | 2 (5.6) | 2 |
| Anemia | 0 (0.0) | 0 | 0 (0.0) | 0 | 1 (11.1) | 1 | 0 (0.0) | 0 | 0 (0.0) | 0 | 0 (0.0) | 0 | 1 (3.7) | 1 | 0 (0.0) | 0 | 1 (2.8) | 1 |
| Hemocytopenia | 0 (0.0) | 0 | 1 (33.3) | 1 | 0 (0.0) | 0 | 0 (0.0) | 0 | 0 (0.0) | 0 | 0 (0.0) | 0 | 0 (0.0) | 0 | 1 (11.1) | 1 | 1 (2.8) | 1 |
|  |  |  |  |  |  |  |  |  |  |  |  |  |  |  |  |  |  |  |
| Psychiatric category | 1 (11.1) | 1 | 0 (0.0) | 0 | 0 (0.0) | 0 | 0 (0.0) | 0 | 0 (0.0) | 0 | 0 (0.0) | 0 | 1 (3.7) | 1 | 0 (0.0) | 0 | 1 (2.8) | 1 |
| Insomnia | 1 (11.1) | 1 | 0 (0.0) | 0 | 0 (0.0) | 0 | 0 (0.0) | 0 | 0 (0.0) | 0 | 0 (0.0) | 0 | 1 (3.7) | 1 | 0 (0.0) | 0 | 1 (2.8) | 1 |
|  |  |  |  |  |  |  |  |  |  |  |  |  |  |  |  |  |  |  |
| Endocrine system diseases | 0 (0.0) | 0 | 0 (0.0) | 0 | 1 (11.1) | 1 | 0 (0.0) | 0 | 0 (0.0) | 0 | 0 (0.0) | 0 | 1 (3.7) | 1 | 0 (0.0) | 0 | 1 (2.8) | 1 |
| Hypothyroidism | 0 (0.0) | 0 | 0 (0.0) | 0 | 1 (11.1) | 1 | 0 (0.0) | 0 | 0 (0.0) | 0 | 0 (0.0) | 0 | 1 (3.7) | 1 | 0 (0.0) | 0 | 1 (2.8) | 1 |

Abbreviation: rHA= recombinant human serum albumin; HSA= human serum albumin; TEAE=treatment emergent adverse events; N= number of subjects analyzed; n=number of subjects with adverse events; m=number of adverse event

**Table S2.** Prothrombin time at baseline and 24 h after infusion

| Group | PT (s) | | eGFR (mL/min/1.73m^2^) | | Ccr (mL/min) | |
| --- | --- | --- | --- | --- | --- | --- |
|  | Screening period (D-14~D-4) | 24 h after the last infusion | screening period (D-14~D-4) | 24 h after the last infusion | screening period (D-14~D-4) | 24 h after the last infusion |
| rHA 10g | 13.5±2.9 | 13.4±2.8 | 102.61±9.15 | 103.18±10.13 | 93.20±7.87 | 94.91±9.80 |
| HSA 10g | 14.8±4.2 | 14.4±3.3 | 108.16±6.90 | 108.05±13.91 | 99.98±11.13 | 99.64±9.49 |
| rHA 20g | 14.2±3.3 | 14.5±2.4 | 105.59±12.35 | 104.92±8.25 | 83.01±7.27 | 81.28±12.29 |
| HSA 10g | 15.7±2.6 | 15.3±2.7 | 106.83±8.98 | 107.15±10.87 | 112.81±6.02 | 113.93±8.21 |
| rHA 30g | 14.6±2.6 | 14.6±3.3 | 103.29±15.19 | 103.23±7.14 | 103.41±11.14 | 103.22±12.92 |
| HSA 10g | 13.4±2.8 | 13.7±2.0 | 103.45±7.95 | 103.70±12.01 | 95.85±9.96 | 96.62±10.28 |

PT: prothrombin time; eGFR: estimated glomerular filtration rate; Ccr: creatinine clearance.

**Table S3. Changes of abdominal circumference (cm) at the end of the main observation period**

|  | **10 g** | | **20 g** | | **30 g** | |
| --- | --- | --- | --- | --- | --- | --- |
|  | **rHA (N = 9)** | **HSA (N = 3)** | **rHA (N = 9)** | **HSA (N = 3)** | **rHA (N = 9)** | **HSA (N = 3)** |
| **baseline** |  |  |  |  |  |  |
| n (missing) | 9 (0) | 3 (0) | 9 (0) | 3 (0) | 9 (0) | 3 (0) |
| Mean (SD) | 96.72 (12.481) | 91.03 (5.254) | 81.36 (4.318) | 90.23 (5.680) | 87.82 (21.184) | 85.97 (4.368) |
| **2 days after the last dose** |  |  |  |  |  |  |
| n (missing) | 8 (1) | 3 (0) | 8 (1) | 3 (0) | 9 (0) | 3 (0) |
| Mean (SD) | 95.53 (10.981) | 84.70 (1.345) | 77.83 (3.794) | 85.60 (6.856) | 85.79 (20.393) | 83.13 (3.661) |
| **Change from baseline** |  |  |  |  |  |  |
| n (missing) | 8 (1) | 3 (0) | 8 (1) | 3 (0) | 9 (0) | 3 (0) |
| Mean (SD) | -0.79 (4.903) | -6.33 (4.028) | -3.66 (3.721) | -4.63 (1.518) | -2.03 (2.499) | -2.83 (0.709) |

**Table S4. Weight changes (kg) at the end of the main observation period**

|  | **10 g** | | **20 g** | | **30 g** | |
| --- | --- | --- | --- | --- | --- | --- |
|  | **rHA (N = 9)** | **HSA (N = 3)** | **rHA (N = 9)** | **HSA (N = 3)** | **rHA (N = 9)** | **HSA (N = 3)** |
| **baseline** |  |  |  |  |  |  |
| n (missing) | 9 (0) | 3 (0) | 9 (0) | 3 (0) | 9 (0) | 3 (0) |
| Mean (SD) | 66.82 (13.600) | 64.20 (6.227) | 55.60 (7.380) | 71.40 (4.513) | 70.73 (9.405) | 68.30 (2.095) |
| **2 days after the last dose** |  |  |  |  |  |  |
| n (missing) | 8 (1) | 3 (0) | 8 (1) | 3 (0) | 9 (0) | 3 (0) |
| Mean (SD) | 65.53 (14.251) | 62.97 (6.463) | 52.88 (6.136) | 69.80 (6.022) | 69.86 (7.850) | 67.87 (2.259) |
| **Change from baseline** |  |  |  |  |  |  |
| n (missing) | 8 (1) | 3 (0) | 8 (1) | 3 (0) | 9 (0) | 3 (0) |
| Mean (SD) | -1.28 (2.655) | -1.23 (0.666) | -3.55 (3.198) | -1.60 (1.808) | -0.88 (2.460) | -0.43 (0.666) |


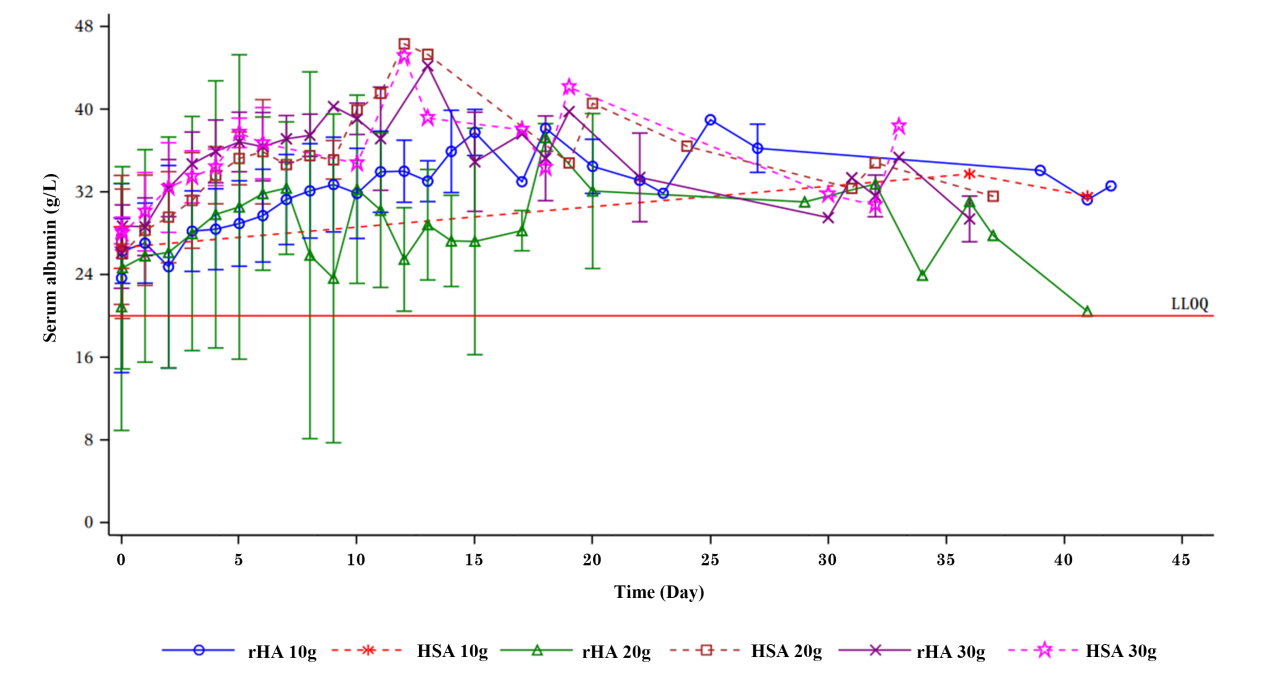


**Figure S1.** Pharmacokinetics profiles of rHA and HSA in patients with ascites due to cirrhosis.


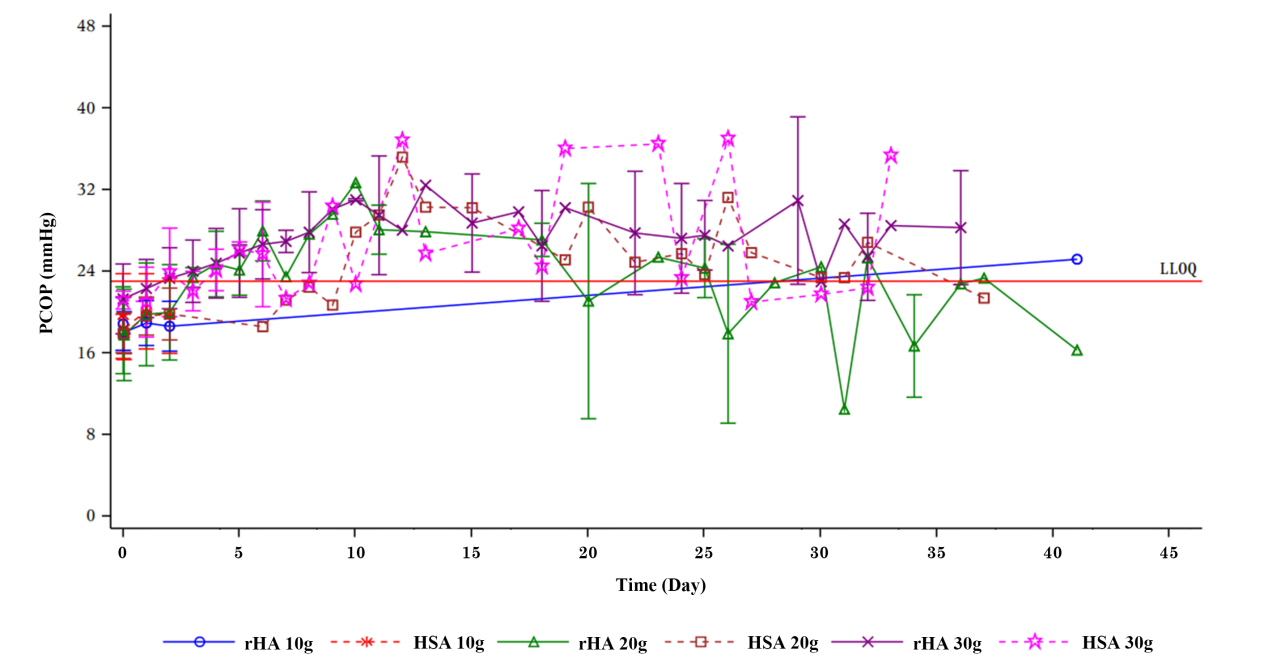


**Figure S2.** Pharmacodynamic profiles of rHA and HSA in patients with ascites due to cirrhosis. Mean PCOP (mmHg) is shown for each cohort.
